# Constant installation of present orientation and safety (CIPOS) - subjective and physiological effects of an ultrashort-term intervention combining both stabilizing and confrontational elements

**DOI:** 10.3389/fpsyg.2022.1035371

**Published:** 2022-11-01

**Authors:** Markus Stingl, Gebhard Sammer, Bernd Hanewald, Franziska Zinsser, Oliver Tucha, Valeska Reichel Pape

**Affiliations:** ^1^Center for Psychiatry and Psychotherapy, Justus-Liebig-University Giessen, Giessen, Germany; ^2^Clinic and Policlinic for Psychiatry and Psychotherapy, University of Rostock, Rostock, Germany

**Keywords:** bilateral stimulation, imagination, EMDR, startle reflex, CIPOS, EMDR-derived technique, dissociation

## Abstract

**Objectives:**

Constant Installation of Present Orientation and Safety (CIPOS) is a Eye Movements Desensitization and Reprocessing (EMDR)-derived technique, which is often used to prepare for the treatment of post-traumatic stress disorder (PTSD). It differs from the latter by involving cyclically recurring exercises in reorientation to the present, interspersed between brief periods of exposure to the traumatic material.

While EMDR is well established as a therapeutic method, the efficacy and mechanisms of action of CIPOS have not been investigated so far. In this pilot study, an experimental setting was used to record the subjective and physiological effects of the CIPOS intervention compared to a control condition with pure mental exposition.

**Methods:**

The study was performed on 30 healthy volunteers aged from 20 to 30 years. Distress was induced using audio files of subjectively stressful situations. Subjective distress was measured *via* the Subjective Units of Distress Scale (SUD), while physiological indicators (noise-induced startle reflex, skin conductance level) were used as measures for objective stress. For each condition, pre- and post-intervention comparisons were calculated.

**Results:**

In both groups, startle reflex potentiation and mean skin conductance level significantly decreased. In the group with CIPOS intervention, but not in the control group, a significant decrease in the SUD value was found.

**Conclusion and significance:**

The results show that the CIPOS technique is as effective as pure mental exposition in reducing physiological stress. In addition, a superiority in reducing subjective distress (indicating a simplified reassessment of the stressful material) was found compared to pure mental exposition. Possible explanations of these effects are discussed.

## Introduction

Before applying trauma-confrontative treatment, the stabilization of patients suffering from posttraumatic stress disorder (PTSD) is often indispensable to prevent retraumatization and worsening of their symptomatology (Cloitre et al., 2011; Cloitre et al., 2012). Even the validated treatment concept of Eye Movement Desensitization and Reprocessing (EMDR), which combines imaginal trauma exposure with bilateral sensory stimuli (Bisson et al., 2013; Shapiro, 1989, 1996, 2002, 2018), implements a preparatory “stabilization” phase in its hierarchical treatment concept. Only if the stability is sufficient, exposure is carried out. This procedure is justified as follows: In patients suffering from PTSD, the ability to actively regulate their emotions is often impaired (Stingl et al., 2021). Therefore, patients tend to avoid unpleasant and potentially overwhelming emotions, especially when being triggered and confronted with traumatic memories (Kumpula et al., 2011). However, this interferes with the required emotional processing of the traumatic experiences (Foa and Meadows, 1997).

Pronounced symptoms of this avoidance phenomenon in PTSD are categorized in the DSM-V as the dissociative subtype of PTSD (American Psychiatric Association, 2013). Dissociation is the partial or complete loss of normal integration of memories, thought processes, action processes, sense of identity, immediate sensations, emotions, or control of body movements (American Psychiatric Association, 2013). In the context of post-traumatic stress disorder, dissociation initially arises peritraumatically. In the traumatic situation, when the victim is no longer able to fight or flight, the affected person “freezes,” and the momentary experience is fragmented, i.e., emotions and bodily sensations are split off from the traumatic content and only partially transferred into the personal narrative. Parallel to distinct memories, amnesias for parts of the trauma exist simultaneously. Later, dissociations can be evoked again by triggers that are similar in content to the traumatizing situation.

Even if comparison outcome studies of patients with or without strong dissociation tendencies show inconsistent effects following trauma-focused therapy, from a clinical perspective dissociation-adapted treatment concepts are necessary: Accompanying the dissociative hypo- or hyperexcitement that impairs confrontational trauma processing, dissociative patients experience recurrent loss of control and feelings of helplessness (as in the traumatic situation). Moreover, dissociation represents a risk factor for re-victimization that must be addressed in psychotherapy in a particular way (Cloitre et al., 1997; DePrince, 2005).

## CIPOS – Constant installation of present orientation and safety

Constant Installation of Present Orientation and Safety (CIPOS) offers a practicable solution to limit the negative impact of dissociation in trauma-focused therapy. This technique, based on the EMDR concept, was designed by Knipe (2010), especially for patients being at risk for dissociation in general and during exposure. Following Knipe (2010), dissociation, or under- or overexcitement, is to be counteracted by two particular components inserted between the very brief exposure to the traumatic material: Firstly, the guided reorientation to the present, and secondly, the reinforcement of the established sense of the present through slow bilateral stimulation. By linking dosed confrontation with targeted reorientation and distancing, stabilization is to be achieved, and traumatic memories are to be processed even in patients with strong dissociation tendencies (Knipe, 2010; Rost, 2014). The underlying theoretical assumptions relate to the universal basic human need to feel safe and secure, as well as the need for orientation and control (Maslow, 1943; Grawe, 2000). These needs are the ones that are severely compromised in PTSD patients due to the traumatic experience itself and the intrusive symptoms that follow, causing the individual to feel recurrently helpless and powerless (Malsow, 1943; Grawe, 2000). There is also evidence of a lack of safety signal learning and an inability to modulate the fear responses with safety cues in PTSD patients (e.g., Jovanovic et al., 2012). CIPOS addresses these issues by continually reinforcing present safety in the therapeutic setting and establishing a constant experience of safety. Furthermore, the recurrent experience of coping with the traumatic memories should make dissociation superfluous as an emotional avoidance strategy and replaces it with active strategies for controlling intrusive experiences.

The detailed process of the CIPOS technique according to Knipe (2010) can be described as follows:

### Preparation

At the beginning of CIPOS, patients are asked to select a subjectively stressful event and estimate how long (1 to 10 s) they can keep this event in their minds. According to Knipe (2010), it is not necessary to narrate the traumatic event in detail but to name its thematic heading. Furthermore, some reorientation techniques are practiced with the patients, which should help them to find their way back to the present after exposure. Examples of such reorientation techniques are given in Table 1.

**Table 1:** Examples of reorientation techniques.

- naming five objects in the room (if reorientation has not yet been achieved followed by the naming of an object not yet perceived before)
- naming five currently perceivable bodily sensations
- move the feet gently back and forth and feel the floor
- counting backwards from 100 in steps of 7
- alternately humming a melody and counting to 10

Note. Other common reorientation techniques such as rolling a hedgehog ball in the hands, catching a ball, or drinking a glass of water were not used due to the electrodes attached to the palm and to avoid motion artifacts.

### Confrontation phase

During the following short imaginative exposure, the patients are asked to keep the stressful situation in their minds. To prevent excessive dissociation at this point, this phase is very short and does not exceed the number of seconds that the patient stated as tolerable before. To anchor the patients in the present, the agreed number of seconds is counted out loud by the therapist.

### Stabilization

At the end of the exposure, patients are invited to take a deep breath and fade out of the memory. The patients are then asked to estimate on a percentage scale (ranging from 0 to 100) how well they can distance themselves from the traumatic material, and to what extent they are reorientated to the present again. If the feeling of distancing is indicated below 95%, the prepared distancing techniques are used. As soon as the patients indicate to feel stable again in the present, their focus is directed to their perception of the present (in emotional, physical, and cognitive terms) and then reinforced with slow bilateral stimulation (BLS). If the feeling of being in the present after the confrontation is immediate >95%, no further distancing techniques are needed, and the feeling of present orientation can be reinforced immediately *via* slow BLS.

After this step, the next confrontation- and stabilization cycle begins which is repeated a maximum of three times.

### The role of bilateral stimulation

The current study focuses on the CIPOS intervention as a whole, i.e., including the element of slow tactile BLS. Even if comparing different stimulation speeds is not the subject of this study, the role of slow versus fast BLS in trauma therapy should briefly be mentioned here. This CIPOS element was taken directly from EMDR therapy. Following the EMDR protocol, BLS can be applied in different sensory modalities using eye movements, rhythmically changing vibrations or tapping (received or performed by the patient), or alternating sounds (Shapiro, 2018). The additive efficacy of BLS has been demonstrated in numerous studies comparing conditions with and without bilateral stimulation. In summary, distress and arousal during imagination of negative scenes can be decreased (Van den Hout et al., 2001; Engelhard et al., 2010; Lee and Cuijpers, 2013; Leer et al., 2014) and attention to positive scenes can be increased (Reichel et al., 2020). The particular effect of BLS seems to depend on the speed of the stimulation: While processing traumatic material is done with rapid eye movements, resource installation is recommended to be done with slow eye movements (Shapiro, 2018). Clinical practitioners report that rapid eye movements promote associative processes that also lead to the processing of further traumatic material (e.g., *via* affect-bridges), whereas slow eye movements - as used in CIPOS - limit this process and therefore are more appropriate to reinforce the existing positive responses. Studies that examined variations in stimulation speed are rare, especially when the target is positive mental imagery or, as in CIPOS, stimulation is intended to reinforce the here-and-now experience following confrontation (e.g., Ichii, 2014). An exception is the study conducted by Amano and Toichi (2016). The authors showed that resource development and installation (RDI) was subjectively more successful when using slow tactile BLS compared to a control group without stimulation. Using near-infrared spectroscopy, they observed significant BLS-induced brain activity, which they associated with the improved recall of more representative pleasant memories, as well as relaxation responses and comfortable feelings. Even if it is not possible to determine the specific effect of slow BLS in CIPOS within this work, this question should urgently be investigated in future studies.

## Measuring the effects of CIPOS – arousal and valence

The CIPOS technique is recommended for stabilization, widely used in therapeutic practice, and taught in EMDR training programs. Nevertheless, there have been no studies on the efficacy or mechanisms of action of CIPOS to date. In the absence of studies of CIPOS itself, studies comparing the pre-post effects of EMDR with other interventions were used as templates (e.g., Ironson et al., 2002; Rothbaum et al., 2005). Emotional distress was measured according to Lang (1995). Following, the emotional experience can be described based on two dimensions: arousal and valence. The arousal dimension represents the intensity of the elicited arousal or excitement. The valence dimension describes the emotional value of something, e.g., “unpleasant or pleasant,” “negative or positive,” or “aversive or appetitive.” The relationship between these two dimensions turned out to be U-shaped: compared to emotionally neutral conditions, arousal is significantly elevated for stimuli with high negative or positive valence (Lang et al., 1993; Lang, 1995). This relationship seems to be because the two poles of the valence dimension are separately associated with the two motivational behavioral systems of avoidance and approach (“behavioral inhibition system,” BIS, and “behavioral approach system,” BAS): when stimuli are evaluated as negative, the avoidance system is activated, and when the evaluation yields a positive valence, the approach system is activated. Since the present study focuses on the increase or decrease of stress reactions, the avoidance component is mainly of interest.

### Physiological arousal measures

#### Electrodermal activity

Several findings indicate that electrodermal activity (EDA) is an index of autonomic sympathetic arousal associated with emotion and cognition.

For example, sounds, music, or film clips that triggered higher arousal led to higher skin conductance, regardless of valence. (Bradley and Lang, 2000; Gomez and Danuser, 2004; Gomez et al., 2005). Other authors also described an increase in skin conductance during the induction of both negative and positive emotions (Bradley and Lang, 2007). In the field of trauma research, various studies have investigated the influence of EMDR interventions on skin conductance as an arousal measure during the confrontation with traumatic content. Aubert-Khalfa et al. (2008) conducted a study with PTSD patients and compared physiological measures in the resting state and during the imagination of traumatic material. After only one EMDR session, a significant reduction in skin conductance was found during the re-imagination of traumatic material compared to the resting state. Wilson et al. (1996) treated patients suffering from various psychological complaints due to traumatic events. They compared EMDR with two EMDR-like treatments, but in the latter bilateral eye movements were not used. Only during the EMDR treatment, but not in the conditions without bilateral eye movements, there was a significant reduction in electrodermal activity, which was interpreted as a relaxation response. Schubert et al. (2010) also investigated the effects of EMDR-typical bilateral stimulation through eye movements in a non-clinical study sample. Compared to EMDR treatment without bilateral stimulation, the group that performed bilateral eye movements showed a significant decrease in skin conductance when confronted with negative autobiographical memories. The authors interpreted this as a decrease in emotional stress.

#### Mean heart rate

Like skin conductance, a change in mean heart rate is associated with emotional arousal during emotional imagination independently of emotional valence (Cook et al., 1991; Fiorito and Simons, 1994). Thus, mean heart rate changes can be used as a measure of changes in emotional states. The influence of EMDR interventions on mean heart rate has been studied in various ways. Sack et al. (2007) compared the physiological parameters of PTSD patients before and after manualized EMDR treatment. After treatment, there was a significant decrease in heart rate during the imaginative confrontation with individually traumatic content compared to neutral scripts. Even after six months, this effect was still present. In the above-mentioned study by Aubert-Khalfa et al. (2008), PTSD patients showed a significant decrease in heart rate during a confrontation with traumatic material after only one EMDR session compared to the resting state. The Sack et al. (2008) group investigated in PTSD patients also changes in heart rate within one EMDR session. They found a significant decrease in heart rate during the session, suggesting an ongoing de-arousal effect caused by the BLS intervention.

### Physiological valence measures

#### Startle reflex

Although skin conductance and heart rate are well-established parameters of emotional responding, they lack an important aspect: they are suitable as measures of emotional arousal, but not for emotional valence. For example, an increased SCL during the confrontation with an affective picture or script can indicate either a pleasant or an unpleasant feeling, but cannot further discriminate them. This problem can be solved by adding the startle reflex paradigm (Bradley and Lang, 2007): The startle reflex is an involuntary blinking response triggered by sudden acoustic stimuli (Lang, 1995; Cuthbert et al., 1996; Grillon and Baas, 2003). As the reflex response increases with aversive stimuli (i.e., simultaneously presented pictures or scripts) and decreases with appetitive stimuli, it is a reliable measure of emotional valence (Cuthbert et al., 1990; Witvliet and Vrana, 1995). As it correlates with brain activity in the amygdalae and other areas of the limbic system, which are hyperactive in PTSD patients (Davis et al., 1993; Angrilli et al., 1996; Lindner et al., 2015), it is neurobiologically consolidated and valid. In a study by Reichel et al. (2020), a significant reduction in affect-modulated startle response was observed in healthy individuals during bilateral tactile stimulation (as used in EMDR sessions). This effect was found for stimulation-only conditions and stimulation during the imagination of negative scripts. Measures of emotional arousal, such as skin conductance response, were not influenced by the therapeutic intervention. In another study by Reichel et al. (2014), adolescents with anorexia nervosa were confronted with pictures of extremely emaciated bodies. While the adolescents reacted subjectively negatively to the pictures, the startle reflex was significantly inhibited, indicating an objectively appetitive response (i.e., positive response). These examples show that the inclusion of the startle reflex provides valuable additional information about emotional responding which exceeds other emotional parameters. The findings presented, especially the ones showing decreases in heart rate, skin conductance, and startle reflex response after only one single EMDR session, support the assumption that changes in physiological measures of arousal can also be expected when intervening with CIPOS.

### Aims and hypotheses

The present pilot study aimed to investigate the subjective and physiological effects of CIPOS on emotional experience in a non-clinical study sample. Emotion induction was achieved of using audio recordings based on individualized affective scripts. Emotional experience was assessed concerning valence (positive vs. negative) and arousal (low vs. high arousal) using subjective and physiological parameters. By comparing an experimental group with CIPOS intervention (including a mixture of very brief exposition and present reorientation techniques) and a control group with continued mental exposition, the following two questions are to be answered: Firstly, is it possible to achieve a reduction in emotional distress *via* CIPOS which is comparable to continued mental exposition? Secondly, if so, is this effect limited to the perception of subjective distress reduction, or is there also evidence on the physiological level (noise-induced startle reflex, skin conductance level)?

## Materials and methods

### Participants

The study sample consisted of 30 healthy subjects (university students) in the age range from 20 to 30 years (SD = 25.5 years). Both sexes (males, n = 8, and females, n = 22) were included. Experimental and control groups were matched for sex, age, and education level. The participants were recruited *via* postings and the university newsletter. All volunteers gave their informed consent and received 15 euros for participation. The local ethics committee approved the study. To exclude currently existing mental disorders, especially trauma sequelae, each subject filed a medical history sheet, the screening form of the DSM-IV (SKID, Wittchen et al., 1997) and the Becks Depression Inventory (BDI-II, German version by Hautzinger et al., 2006). People with documented severe mental disorders (drug abuse, dementia, schizophrenic psychosis, and intellectual disability), neurological diseases (such as seizures in the medical history), severe hearing or visual disabilities, medication with influence on startle reflex response (benzodiazepines, buspirone, and opioids), recent medication switch within the last 2 weeks, or insufficient knowledge of the German language were not included to the study. If subjects met the inclusion criteria, their imaginative ability was assessed by using the Questionnaire upon Mental Imagery (QMI; Sheehan, 1967; German version by Löffel, 1994) and the extent of dissociativity using the Dissociative Experience Sale (DES by Bernstein and Putnam, 1986; German version FDS by Spitzer et al., 1995).

### Procedure

#### Preparing stimulus material

**Figure 1:**
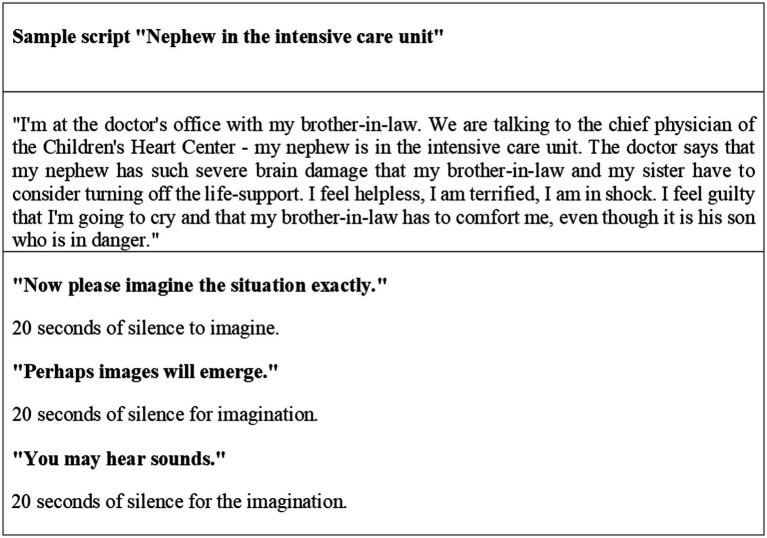
Individualized sample script from a female subject in the experimental group with the caption “Nephew in intensive care.”

In the next step, the emotion-inducing stimulus material based on autobiographic distressing events was prepared. Individual scripts (each describing a specific situation) were created and then presented *via* headphones. To prevent decreasing concentration over the course of the experiment, the total number of scripts per subject was limited to four. After choosing the situations and finding headlines, the subject was then asked to judge them on the Subjective Units of Distress (SUD) scale (ranging from 0 = no distress to 10 = maximum possible stress). Situations eliciting a distress score of at least 5 during mental imagery were included in the study. Situations representing traumatic events such as experiences of violence were not included. The following situations were mentioned most frequently: deaths (20), illness (19), accidents (8), stressful professional or university situations (16), stressful interpersonal situations such as separations and arguments (48), and other fearful situations (9). Based on the first two parts of the SORCK schema (Kanfer and Saslow, 1974) - situation and organism -, a precise analysis of the situation followed: Firstly, it was asked what exactly had happened in the situation and secondly, if and which sensory impressions (e.g., certain noises or smells) were witnessed. Thirdly, it was asked what thoughts came up, and at last, if and which feelings were experienced. Based on this information, four scripts were created, which were edited by the subject and then recorded on audio files. It was ensured that the resulting scripts were comparable in terms of form and content criteria. An example script is shown in Figure 1.

#### Preparing reorientation techniques (only CIPOS group)

This step was performed exclusively for the participants of the CIPOS group. Each group participant was invited to try out several reorientation techniques from a list which was based on various sources on EMDR (Huber, 2009; Knipe, 2010; Rost, 2014; Böhm, 2016), see Table 1. Listing of five objects in the room (if reorientation has not yet been achieved: listing an object that has not yet been perceived), 2. Listing of five body sensations, 3. Gently moving the feet back and forth on the floor, 4. Calculating backward from 100 in steps of 7, and 5. alternately humming a melody and counting to 10. Techniques that interfered with the physiological measurements were ruled out (e.g., the technique of rolling a hedgehog ball in the palms). Those techniques that the participant experienced most effectively were used in the study.

#### VAS before the experiment

Directly before testing, mood (ranging from 1: positive, to 100: negative) and arousal (ranging from 1: low arousal, to 100: high arousal) were assessed *via* Visual Analogue Scales (VAS).

### Experiment

Each participant was placed on a chair about 50 cm in front of a computer screen, and electrodes, headphones, and vibration pads were attached. The computer screen served to present a fixation cross at which the test subjects should direct their focus. After this preparation, the presentation of the first audio recording with the first script (i.e., situation) began.

#### Pretest

Script presentation was done aurally *via* headphones. Each audio recording consisted of four parts: “baseline” (3 startle tones), “hearing” (1 startle tone), “imagination” (1 to 2 startle tones), and “rating.” Following the guidelines of Blumenthal et al. (2005), one or two startle tones were presented at the beginning, which were not included in the evaluation. After a 20-s baseline part with nothing to hear than the three startle tones, the first script was presented, divided into two parts: “hearing” and “imagination.” In the “hearing” part, the situation was described for about 40 s, and one startle tone was applied.. The “imagination” part was initiated with the request “Please imagine the situation now exactly.” Further instructions followed to visualize the situation as vividly as possible (“Perhaps pictures will appear,” “Perhaps you will hear noises,” and “Perhaps thoughts or feelings will arise”). In this phase, additional one to two startle tones were presented. At the end of imagination, the test person was asked to fade out (“Fade out now.”) and to assess the subjective stress during exposure (“SUD rating: “How stressful was this situation on a scale from 0 to 10?.” An exemplary script of a participant from the experimental group can be seen in Figure 1.

**Figure 2:**
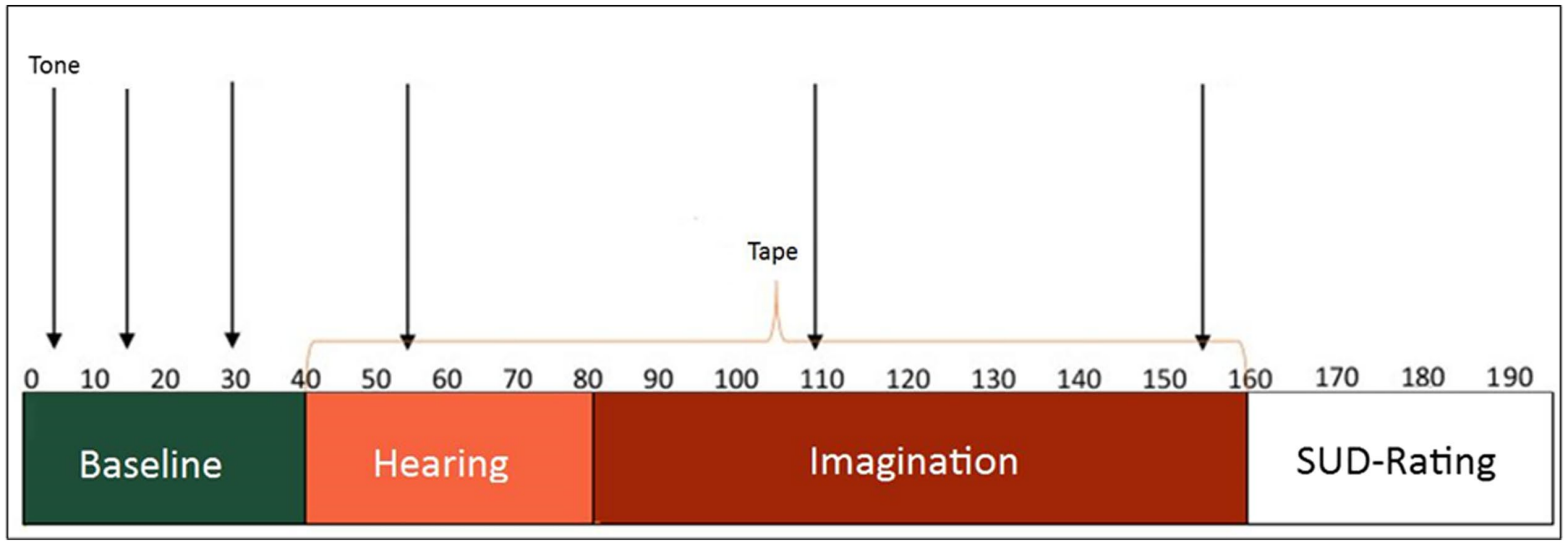
Trial sequence. Sample timeline of the Habituation, Pre-exposure, Hearing, and Imagination. The times at which the startle tones sounded are indicated by the black arrows. Each participant completed three different trials.

During the entire measurement period, beginning with the baseline, five to six startle tones were applied. Based on Fanti et al. (2017), who recorded the startle reaction and skin conductivity while viewing video sequences, the tones were played 10, 11, or 15 s after the beginning of the respective part. To reduce the risk of habituation effects, the tones per test session appeared at different times, whereby one of three different combinations was used in each case. In addition, one or two startle tones were presented right at the beginning of the measurement phase, which were not included in the evaluation. Figure 2 shows an example of a timeline for the first of the three combinations.

#### Intervention

After the pretest, the intervention phase was initiated, which differed depending on the respective group.

In the *experimental group*, a shortened form of the CIPOS intervention by Knipe (2010) was used. At first, the subject was asked to rate her/his current sense of the present in percentage (ranging from 0 to 100%), using the phrase: “Perhaps there are still remnants of memory. To what percentage are you in the here and now?.” If the sense of present was below 95%, one of the prepared reorientation techniques was used. Afterward, the sense of present was inquired again. If the sense of present was at least 95%, or until a maximum of three present reorientation trials, the achieved sense of present was reinforced using bilateral tactile stimulation. Tactile stimulation was chosen to exclude possible artifacts due to visual stimulation during recording of the eye blink reflex *via* muscular signal recordings.

The participant was asked to pick up the provided vibration pads and to focus on the sense of the present.

During the mental focusing, five vibration sets were applied to the palms of the hands, alternating from left to right. According to Ichii (2014) we quantified the speed of the slow alternating bilateral stimulation at 0.2 Hz. Therefore, each vibration set on one hand lasted 5 s. We chose a middle intensity according to the scale of the device. Tactile vibration stimulation was indexed with 5×3.5×1 cm plastic pulsers of the Deluxe Tac/Audioscan Device Revision 5.1 from NeuroTek stimulation (Contact Neurotek Corporation; Wheat Ridge, CO).

In the *control group*, the test persons were asked to stay mentally in the situation. This was done using different, slightly varying sentences like “Continue to imagine the situation” or “Continue to imagine what happened.” This way, a comparable cognitive strain, and attention should be generated by the experimenter as in the experimental group. The participants were matched with the participants of the experimental group with regard to the times for the intellectual exposure: They imagined the situation as long as their counterparts from the experimental group had needed for the reorientation. In the control group, no BSL were applied.

#### Post-test

After the intervention, the same script was presented *via* headphones again. The procedure (i.e., baseline, hearing, imagination, and subsequent rating) was carried out in the same way as the pre-test. After a one-minute break, the pre-test of the next script was initiated.

#### VAS after the experiment

As the last step, mood (ranging from 1: positive, to 100: negative) and arousal (ranging from 1: low arousal, to 100: high arousal) were assessed again *via* Visual Analogue Scales (VAS).

### Electromyographic activity

The Lab Linc V Tower from Coulbourn Instruments recorded the physiologic responses and generated the startle tone (contact Coulbourn Instruments; Holliston, MA). The eyelid closure component of the startle reflex was measured as an electromyogram (EMG) of the left orbicularis oculi muscle with Ag/AgCl miniature electrodes. A 95-dB white noise of 50 milliseconds was generated by the V85-04 Audio Source Module and presented binaurally through headphones. After sampling at 1000 Hz and filtering through a 30–500 Hz isolated bioamplifier with bandpass filter model V75-04, raw EMG signal was rectified and integrated with a time constant of 20 ms. The integrated EMG signal was digitally analyzed for magnitude and latency to peak using Coulbourn Instruments’ Human Startle Reflex System HMS 500 software. EMG “magnitude” was defined as the difference between peak EMG (highest EMG value within 20 to 150 milliseconds after the noise) and baseline EMG (EMG value within the last 100 milliseconds before) in accordance with Blumenthal et al. (2005). Trials that showed a lack of reflex response, an EMG magnitude <0.1 μV, or a latency to peak >150 milliseconds were categorized as non-response and set to 0 μV. Trials with latency to peak <20 milliseconds, motion artifacts, or excessive baseline activity were categorized as miss.

Participants were categorized as non-responders if their null responses or misses accounted for more than one-third of all recorded trials.

### Electrodermal activity

Electrodermal activity (EDA) was derived *via* Ag/AgCl standard electrodes on the hypothenar muscle of the nondominant hand. The signal was recorded using the Brain Products GmbH V-Amp 16 amplifier with a voltage across the electrodes of 0.5 V (Contact Brain Products GmbH, Gilching, Germany). Raw arbitrary data were processed using Ledalab software (Benedek and Kaernbach, 2010).

The EDA slope was digitally deconvolved by the general response shape which results in a large increase in temporal precision. The data was decomposed into continuous phasic and tonic components (Benedek and Kaernbach, 2010). Skin conductance level (SCL) was defined as the mean tonic activity of decomposed tonic component in the defined response window (900 to 4,000 milliseconds after trigger). The triggers were placed at a sufficient distance to the last startle tone to prevent confounding with the tones. 0.01 muS was used as the minimum amplitude threshold. Responses with motion artifacts or excessive baseline activity were defined as misses. Participants with electrodermal non-responses (i.e., maximum SCL equal to zero) were excluded from further analysis.

### Data reduction and analysis

The collected data were statistically processed using IBM SPSS Statistics 22.0 Software. The raw data of each subject were averaged per script (script 1 to 4), part (baseline, hearing, imagination), and phase (pre vs. post-intervention). The normal distribution requirement was checked by using the Kolmogorov–Smirnov test. EMG data was range-corrected. To avoid confounding with the startle noise, EDA data were analyzed only for non-startle trials. The data of the first “baseline” part was used to control for group differences in physiological baseline parameters (general startle response, general SCL) before the experiment. It was ensured that physiological changes in the course of the experiment (such as script position or baseline changes over time) did not have any influence on the measured effects (see Results section). Group differences in relevant baseline variables such as age, sex, pre-test mood, pre-test arousal, scores of the screening questionnaires, startle reflex response, and SCL were tested by using independent t-tests.

In the next step, analyses of variance (ANOVA) for repeated measures were calculated. At this point, “hearing” and “imagination” data were averaged as there were no significant differences between them (see Results section). ‘Phase’ (pre vs. post-intervention) was used as a within-group factor and ‘group’ (CIPOS vs. control condition) as a between-group factor. As dependent variables, SUD value, startle reflex magnitude, and skin conductance level were used.

For all analyses, value of *p*s < 0.05 (two-sided) were considered statistically significant. In cases where Bonferroni corrections for multiple measurements were necessary, the calculated value of p was multiplied by the number of measurements. In cases in which sphericity could not be assumed, the Greenhouse Geisser correction for degrees of freedom was used. Contrast analyses were performed for the empirically well-founded hypotheses on general emotional reactivity. In all other cases, post-hoc tests were used.

## Results

### Sample characteristics

Groups did not differ significantly in age, sex, and education level, and also not in the scores of the screening questionnaires. In the QMI, subjects in both groups showed good overall imaginative abilities. The level of dissociativity in the FDS was well below the cut-off score for a clinically relevant dissociative disorder; likewise, according to the BDI-II, the level of depressiveness was below the clinical cut-off score for depression. There were no significant group differences in pre-test mood [t (28) = 0.53; *p* = 0.600] and arousal [t (25.5) = 0.08; *p* = 0.939] nor in the physiological basic parameters, i.e., in the startle magnitude [t (15) = −1.79; *p* = 0.094] and the SCL [t (7) = 0.57; *p* = 0.584].

One individual from the experimental group and one individual from the control group were classified as EDA-Nonresponders. There were no EMG-Nonresponders in both samples, but the data of one subject had to be classified as missing because of artifacts. A notable problem was EDA data loss in the baseline period because of technical problems. The data of one subject in the pre-test baseline and >2/3 of the subjects in the post-test baseline had to be excluded, which is why an evaluation was not possible for this data in a meaningful way. For the other time periods, no exclusion of subjects because of technical problems or artefacts was necessary.

Demographic data, clinical sample characteristics, the results of the VAS pre-test ratings, and physiological baseline parameters (startle magnitude and SCL) are summarized in Table 2.

**Table 2:** Demographic and clinical sample characteristics before the experiment.

	CIPOS Group (EG)		Control Group (CG)		EG vs. CG
	*M*	*SD*	*M*	*SD*	*p*
Age	24.7	2.6	24.3	1.9	0.638
BDI	6.2	3.5	10.3	8.9	0.117
FDS	8.0	4.7	10.6	8.4	0.298
QMI	2.6	0.7	2.3	0.4	0.202
VAS_Mood_	32.3	13.4	35.0	14.1	0.600
VAS_Arousal_	39.6	24.8	36.3	27.1	0.939
Startle_Baseline_	19.7	8.7	22.6	9.3	0.094
SCL_Baseline_	62.7	12.3	55.9	0.7	0.584

Values are means (M) and standard deviations (SD). Subjective basis parameters were measured before the experiment via visual analoge scales for mood (reaching from 1, pleasant, to 100, unpleasant) and arousal (reaching from 1, low arousal, to 100, high arousal). Physiological basis parameters included range corrected startle reflex (in mV) and skin conductance level (arbitrary data) during the first baseline phase. Group comparisons were done via one-way ANOVAs (two-tailed). Where necessary, Welsh correction for unequal sample sizes was used. Experimental group (CIPOS) and control group with prolonged exposition (CG) did not differ significantly in subjective and physiological basis parameters before the testing.

### Subjective units of distress

The overall analysis revealed a significant interaction effect between phase (pre vs. post-intervention) and group [CIPOS vs. control condition; *F* (1,28) = 5.96, *p* = 0.021). In the CIPOS-group, the SUD value decreased significantly from pre to post [*F* (1,14) = 10.85; *p* = 0.005]. In the Non-CIPOS-group, no significant changes of the SUD value were found [*F* (1,14) < 1; *p* = 0.487]. The main effect for phase was also significant [*F* (1,28) = 10.14, *p* = 0.004], whereas the main effect for group was not [*F* (1,28) = 3.83, *p* = 0.060].

### Physiological measures

#### Skin conductance level

**Figure 3:**
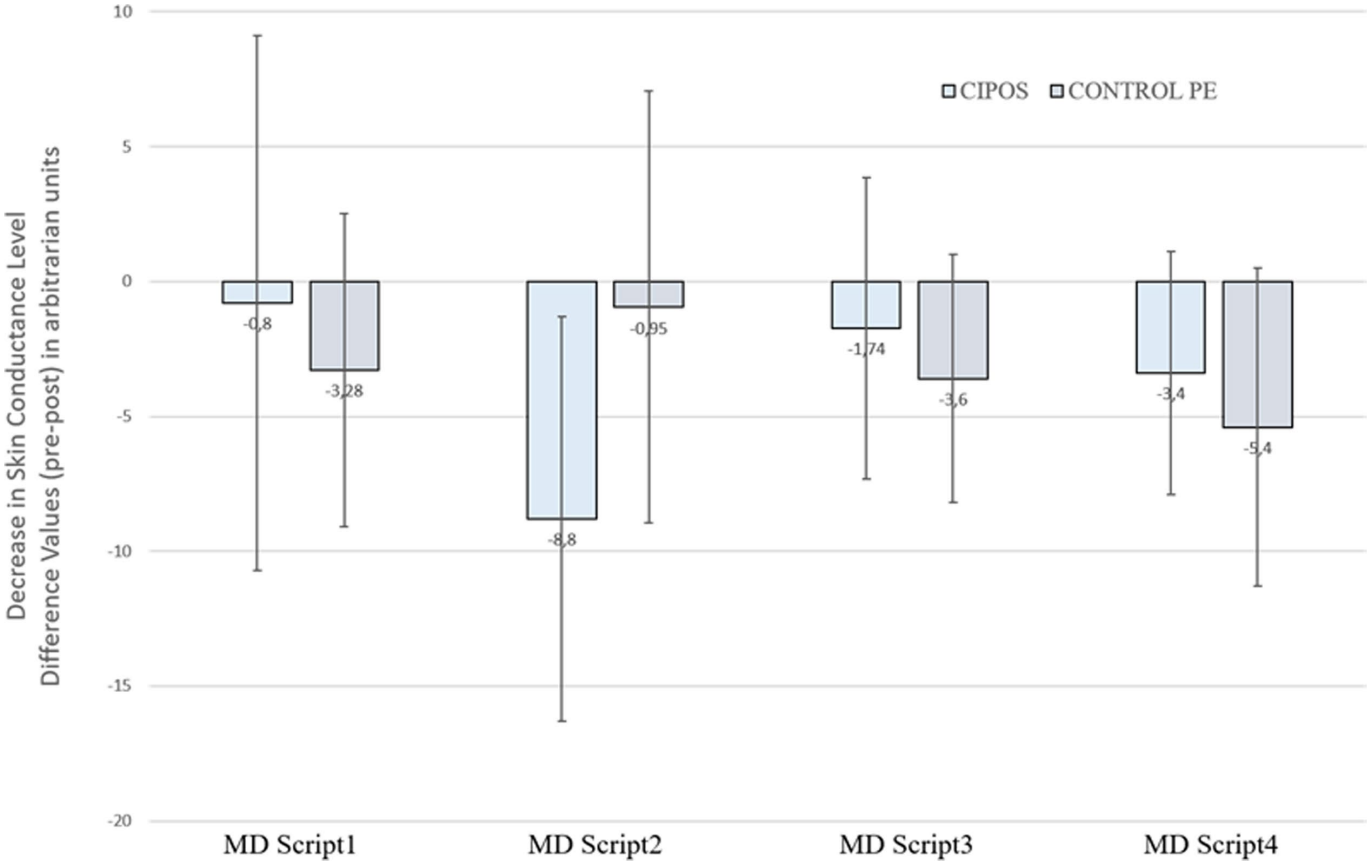
Interaction between Script Position, Phase, and Group in SCL. Note. Exploratory interaction effect between script position (script 1 to 4), phase (pre- vs. post-intervention), and group (CIPOS vs. Control with prolonged exposition) for the decrease in SCL. Values are pre-intervention to post-intervention differences. SCL = skin conductance level.

Differing between “hearing” and “imagining” the scripts had no main effect on the SCL [*F* (1,26) < 1, *p* = 0.423] and did not interact with phase [*F* (1,26) = 2.77, *p* = 0.108] or phase and group [*F* (1,26) < 1, *p* = 0.372]. Therefore, the SCL data was averaged over these two parts (i.e., “hearing” and “imagination”). A significant main effect for phase [*F* (1,26) = 6.89, *p* = 0.014] was observed: SCL decreased significantly from pre to post in both groups. The main effect for group [*F* (1,26) = 6.89, *p* = 0.0176] as well as the phase and group interaction effect [*F* (1,26) < 1, *p* = 1.00] was not significant (see Figure 3).

#### Startle magnitude

Similarly to the SCL, the startle reflex data of the “hearing” and the “imagination” part was averaged, as there was no main effect [*F* (1,28) < 1, *p* = 0.460] and no interaction effects neither for phase [*F* (1,28) = 2.915, *p* = 0.099] nor for phase and group [*F* (1,28) < 1, *p* = 0.819]. A significant main effect for phase [*F* (1,28) = 6.702, *p* = 0.015] was found: Startle magnitude decreased significantly from pre to post in both groups. The main effect for group [*F* (1,28) = 3.687, *p* = 0.065] and the phase x group interaction effect [*F* (1,28) < 1, *p* = 0.440] were not significant.

### Visual analogue scales

To check the persistence of the positive subjective effects found for the CIPOS intervention, mood and arousal before and after the (whole) experiment were recorded in both groups *via* VAS.

For this purpose, ANOVAs with “VAS mood” respectively “VAS arousal” as dependent variables were performed with group (CIPOS vs. control condition) and time (before vs. after the experiment) as independent variables.

#### VAS arousal

For subjective arousal, no significant interaction effect for time and group [*F* (1.28) = 2.05; *p* = 0.163], no main effect for time [*F* (1.28) = 2.91; *p* = 0.099], and no main effect for group [*F* (1.28) = 1.21; *p* = 0.280] was found. The subjective arousal thus did not change significantly in both groups over the course of the experiment.

#### VAS mood

In contrast, a pre-post vs. group interaction effect was found for subjective mood [*F* (1.28) = 3.93; *p* = 0.057]: Whereas mood did not change significantly from the beginning to the end of the three trials in the CIPOS group [*F* (1,14) < 1; *p* = 0.343], controls with pure mental exposition reported that their mood became significantly worse [*F* (1,14) = 23.39; *p* < 0.001]. A marginal interaction effect was found (*p* = 0.057). After adding imagination ability as a covariate, the effect became significant [*F* (1.27) = 6.118; *p* = 0.020]. Main effects for the pre vs. post intervention [*F* (1.28) = 12.88; *p* = 0.001] and group [*F* (1.28) = 5.75; *p* = 0.023] were significant even before covariant analysis.

A summary of the values of all observed parameters before and after the intervention for the CIPOS and control groups can be found in Table 3.

**Table 3:** Subjective and physiological parameters pre and post intervention of the CIPOS group and the Control group (CG) with prolonged exposition.

	CIPOS group *M (SD)*			Control group *M (SD)*		
	Pre	Post	p	Pre	Post	p
SUD	5.7 (1.7)	4.9 (1.7)	0.203	6.3 (1.1)	6.2 (0.9)	0.487
SCL	68.2 (23.9)	65.9 (26.6)	0.134	55.1 (24.1)	52.8 (25.5)	0.039
Startle	0.46 (0.15)	0.45 (0.16)	0.310	0.56 (0.15)	0.54 (0.14)	0.203

Values are means (M) and standard deviations (SD). Subjective distress was measured with the Subjective Units of Distress Scale (ranging from 0, no distress, to 10, maximum distress). Physiological parameters included range corrected startle reflex (in mV) and skin conductance level (arbitrary data). Pre vs. post intervention comparisons were done via one-way ANOVAs with repeated measurements for each group (two-tailed).

The effect sizes [Cohen’s d; (a d value of 0.2 represents a small effect size, d = 0.5 represents a medium effect size, and d > 0.8 represents a large effect size)] of group differences illustrate the strong decrease in SUD values in the CIPOS group compared to the control group with prolonged exposure (Cohens d = 0.992) Only small group differences (with small advantages of the CIPOS intervention) are observed in terms of the decrease in startle magnitude (Cohens d = 0.286), while the same effects can be found in the decrease in SCL (Cohens d = 0.000).

### Potential confounders

#### Script position

To examine whether the position of the script had any influence, repeated ANOVA tests were performed on phase (pre vs. post-intervention) and script position (script 1 to 4). This was done for both the subjective and the physiological data.

For the SUD value, the main effect for script position was significant [*F* (3,84) = 4.54, *p* = 0.005], and a linear trend was observed [*F* (1,28) = 10.22, *p* < 0.003] with significantly increasing SUD values from script 1 to script 4. However, this effect did not influence the intervention effect: The interactions between script position and phase [*F* (2.14, 59.92) = 1.16, *p* = 0.323], script position and group [*F* (3,84) < 1, *p* = 0.836], as well as script position, group and phase [*F* (3,84) < 1, *p* = 0.694] were not significant.

On the physiological level, script position did not interact with the startle magnitude (main effect: *F* (3,69) < 1, *p* = 0.329], and the interaction effects for script position and phase [*F* (3,69) < 1, *p* = 0.838], script position and group [*F* (3,69) < 1, *p* = 0.893], and script position, group and phase [*F* (3,69) = 1.956, *p* = 0.129] were not significant. For the skin conductance level, also no significant main effect for script position was found [*F* (3,69) < 1, *p* = 0.478], and there was no interaction between script position and phase [*F* (3,69) = 1.274, *p* = 0.290] or script position and group [*F* (3,69) < 1, *p* = 0.743]. Confounding the data by noise-related habituation effects could thus be excluded. However, a significant script position, group, and phase interaction effect was found [*F* (3,69) < 1, *p* = 0.008]: In the CIPOS group, a strong initial SCL-reduction was observed (script 1 to 2), followed by a slow increase (script 2 to 4). In the control group, in contrast, the SCL initially increased (scripts 1 to 2), followed by a slow decrease until the end of the experiment (scripts 2 to 4). These side results are explorative and should be investigated further.

#### Baseline effects

Additional control was realized by checking baseline effects over time for the physiological data. Because of data loss in the EDA data files, this was done for the EMG data only. For the EMG baseline data (startle magnitude), a significant main effect for phase (pre vs. post intervention) was observed [*F* (1,28) = 6.944, *p* = 0.014]: The startle magnitude decreased significantly from pre to post. However, this “before intervention” effect was comparable in both groups [main effect for group: *F* (1,28) < 1, *p* = 0.093], and no interaction effect between phase (pre vs. post intervention) and group [*F* (1,28) < 1, *p* = 0.624] was found.

## Discussion

This study examined the subjective and physiological effects of CIPOS and an exposition-only condition using subjective and objective measures. The data shows that physiological arousal significantly decreased from pre- to post-intervention both for the experimental group (CIPOS) and the control group (continued exposition), meaning that a comparable reduction in startle reflex and skin conductance level was found. Additionally, a significantly better reduction in subjective distress was observed: Only the CIPOS group reported significant reductions in SUDs from pre to post than the exposition-only group at the end of the experiment. Further, a significantly less negative mood was found at the end of the experiment for the CIPOS group (compared to the beginning of the experiment). These results are unexpected and therefore require immediate further analysis. Thereby, three questions should be in focus:

### How can the physiological arousal decrease in both groups be explained?

The time of imaginative exposure in CIPOS is much shorter than in the exposition-only condition and even shorter compared to the reprocessing phase in the standard EMDR procedure. Following the habituation model, this shortening of the exposition should *prevent* the habituation process and therefore prevent effective processing of the traumatic experience. Nevertheless, in the present study a comparable physiological arousal decrease in both conditions (CIPOS and prolonged exposure) was found. Even if habituation to the stressful audio files cannot be completely ruled out as an explanation, there are various indications in the literature that CIPOS-like interventions are actually physiologically effective: Several studies to date have shown that recurrent short and intermittent exposure sets, combined with BLS used in the standard EMDR procedure, are in general as effective in reducing traumatic stress as established treatment approaches like Cognitive Behavioral Therapy (CBT) or prolonged exposure. The underlying working mechanisms of these short exposure-BLS effects cannot be explained using the classical habituation model and are the subject of an ongoing debate. As alternative explanations the following models are discussed: the “orienting response hypothesis” (Armstrong and Vaughan, 1996), “the working memory model” (Baddeley and Hitch, 1974; Andrade et al., 1997), and the activation of specific neuronal pathways contributing to fear extinction (Baek et al., 2019; Rousseau et al., 2019). These models are in part capable of explaining potential physiological effects of CIPOS: For example, following the orienting response hypothesis, drawing attention to the present should induce an intensified orienting response. This should be depicted by an increase in sympathetic activity, followed by a parasympathetic response, as found in this study. An alternative explanatory approach is the *working memory model.* This hypothesis is based on the assumption of a limited working memory capacity. Repeated present orienting exercises may tax working memory resources and alter the quality of the stressful imaginations. To prove this preliminary assumption, the specific workload of this kind of dual-task should be further tested to determine its taxing power on working memory.

### How can the superiority of CIPOS to prolonged exposition on the subjective level be explained?

The CIPOS-focus is explicitly resource-oriented as the client is actively guided to perceive the resource of present safety, which is reinforced by slow BLS after exposure. In general, resources are any abilities that help people to reach their goals or to overcome impediments (Leeds, 2009; Gross et al., 2012), and can be used to induce a positive affective state. Repeated immersion in this, as is achieved in CIPOS, seems to provoke a significantly larger emotional relief. This could only be shown on the subjective level, which may indicate that this effect is primarily cognition-based. By changing between very short exposure and present-orientated exercises, a cognition reorientation process may be induced, for example by changing between cognition such as “I am not safe” versus “I am safe.” This cognitive reorientation process may enable PTSD patients to cope with their negative experiences in the past: One of the main problems with PTSD is that sufferers are often unable to adequately distance themselves from the stressful memories and locate them in the past. The memories therefore often have a present-tense quality, which in turn contributes to the perpetuation of the symptoms. CIPOS’s focus on consciously staying in the present, as a contrast experience to the stresses of the past, may provide an important resource for coping with traumatic experiences. This guided focus may serve as a specific offer of adaptive information to help the clients distance themselves from their past experiences. In consequence, clients may be able to regulate their emotions in a better way as the observed changes in arousal and valence indicated. Moreover, clients may achieve strengthened self-efficacy instead of renewed helplessness against thoughts of the past.

### How can the interaction effect between script position, phase, and group (CIPOS vs. prolonged exposition) for the decrease in SCL be explained?

In the prolonged exposition group, the initial increase of the SCL was followed by a slow decrease until the end of the experiment. As the SCL measures the tonic phase of the EDA, this can be interpreted as a changing process starting with an initial stress response towards an ongoing habituation response over the experiment. This confirms the assumption that the effects of the pure mental exposure condition are due to an initial orienting response followed by habituation.

In contrast, within the CIPOS group, a first decrease in SCL was observed. This supports the above-mentioned assumption that the short exposure combined with an active resource orientation can cause a rapid decrease in (also objective) stress. In the course, however, it becomes apparent that the objective stress level increases again after the second script has been processed. Accounting to the thesis, that during the short exposition within the CIPOS phase only reminders of the distressing memories are kept in mind, the ongoing procedure of repeated exposition may guide the individual to focus more on the specific distressing contents and counteracts the postulated more superficial processing. Based on these findings, it makes sense that in clinical practice only one distressing situation is dealt within a single CIPOS session.

As the last reported side results are explorative ones, they should be investigated further.

Taken together, the CIPOS procedure differs fundamentally in two respects from prolonged exposure: Firstly, the time of imaginative exposure without BLS in CIPOS is much shorter than in the exposure condition and even shorter compared to the reprocessing phase in the standard EMDR procedure. Secondly, the CIPOS-focus is explicitly resource-oriented as the client is actively guided to perceive the resource of present safety, which is reinforced by slow BLS after exposure. According to these two distinctions, the CIPOS procedure can be subdivided into two major parts (without the preparation of the reorientation techniques). The first part is to bring the stressful memory to consciousness and to intensively activate it in the imaginative confrontation. The second part consists of explicit resource activation *via* active reorientation. Looking at the timing of the two parts, it is obvious that reorientation occurs at a time when the stressful experience is still activated and receptive to change. Various studies have shown that recalling memories can briefly place them in an unstable state, updating memory and enabling learning (e.g., Nader and Hardt, 2009). Based on this mechanism, interventions applied in this vulnerable state can alter traumatic memories and reduce intrusions (James et al., 2015; Hagenaars et al., 2017).

A recently new development in trauma therapy, the Flash Technique (FT; Manfield et al., 2017, Manfield and Engel, 2018), also combines very short exposure with focusing on a positive experience. Here, patients are asked to identify a memory that is stressful and needs to be worked on, and then to focus on a positive experience. At the verbal prompt “flash” patients should recall the memory and immediately return to the positive memory or blink their eyes three times when prompted (Manfield and Engel, 2018). One goal of this approach, similar to CIPOS, is to enable patients to reduce their experience of distress in the short term and prepare them for more extensive confrontational trauma treatment such as EMDR. The results of this procedure in reducing subjective distress are promising (Manfield et al., 2021), even in dissociative patients as case studies indicated (Shebini, 2019; Wong, 2019).

To explain the effects of FT in PTSD, Wong (2021) proposes an elaborated model founded on an extrapolation of working memory and neurobiological research. Summarizing the model, the very brief access to the traumatic memory maps in the working memory is only a “reminder of the memory” and prevents the overactivation of the amygdalae. Together with the setting of a positive engaging focus, patients can remain calm while they access the traumatic memory, establishing a prediction error that is a prerequisite for further processing and reconsolidation. Subsequently, the prompted blinks support continuation of this process. Although studies confirming the presumptions of the model are still pending, it offers a plausible alternative starting point to explain the effects of short imagination combined with a resource-guided focus. The results of this study are compatible with the main features of the presented model. The physiological data confirms a decrease in arousal associated with a positive change in valence following short exposure without the in-depth and detailed engagement of the memory and guided positive focusing. The subjects appear to be significantly more relaxed over the course of the experiment conducted in this study, generating the condition to create further consolidation processes due to CIPOS.

## Implications

This work is to be understood as a basic study of the CIPOS technique which aims to increase the state of knowledge about the mechanisms of action of this therapeutically widely used method. Contrasting CIPOS with an exposition-only condition in a non-clinical study sample, changes in emotional reactivity during the confrontation with personal distressing scripts were investigated. Therefore, emotional valence and arousal changes during imagining the scripts were recorded. In addition to subjective measures, objective physiological measures (startle reflex as valence measure, SCR as arousal measure) were also recorded.

The results show that it is principally possible to reduce stress in healthy individuals *via* CIPOS and that this effect can be compared to an impact induced by prolonged exposition. This is accordant to the suggestion of clinicians that short exposure, combined with resource-oriented focusing on the present safety, facilitates the emotional processing of the targeted memories. In contrast to the mere exposure condition, clients evaluated their subjective distress reduction *via* CIPOS significantly better. These findings are promising and suggest that trauma exposition, as implemented by CIPOS, can be tolerated also by heavily burdened and highly dissociative patients. The strong subjective relief also suggests that this method is capable of preparing clients for a subsequent more confrontational trauma therapy.

## Limitations

Nevertheless, the study has some limitations. At first, it was performed with small sample size, and the witnessed effects were comparably low. The data was collected in a student sample and is restricted to healthy individuals in a certain range of age (20 to 30 years of age). The study shows the effects of CIPOS in a lab setting, but not necessarily the effects of the CIPOS intervention in ongoing trauma therapy. The emotional processing of distressing memories in healthy and traumatic memories of PTSD patients differs substantially: Resulting from the overactivation of the amygdalae, emotion inhibition abilities in patients with PTSD are frequently disturbed (Etkin and Wager, 2007; Terpou et al., 2019). Due to this reason, the findings cannot be transferred to patients suffering from PTSD or who are highly susceptibility to dissociation. Although the scripts were personalized and subjectively had a SUD > 5, memories of traumatic events were not part of the study. This could have contributed to the missing SCL increase after presentation of the stressful audio files. An alternative explanation for this problem could be data loss and physiological over-arousal in the baseline phase, which may have masked an SCL increase during script presentation. Adding a relaxation phase before starting the measurement could be helpful here. The study focuses on short-term effects only. Therefore, no statements can be made about the stability of the effects. Moreover, the study does not distinguish between single components of the intervention. For example, no differentiation is made on whether present orientation is effective although without reinforcing *via* BLS. To enhance present orientation, tactile stimulation was used. Even if tactile stimulation has been shown to be comparable to visual stimulation in previous studies (Nieuwenhuis et al., 2013), the present results are not automatically generalizable to non-tactile stimulation types. The inclusion of other measures of distress such as corrugator EMG, heart rate, or cortisol level is also advisable and could be applied to validate the results. Finally, the absence of a non-intervention group is a notable problem: Whereas noise-related habituation effects can be excluded (see subsection “Confounders”), habituation to the audio-files cannot be ruled out at this point of time. This means that the post-interventional decrease in startle magnitude in both groups could only be the result of habituation effects. However, this seems unlikely, since exposure-based interventions were shown to decrease the affect-related startle reflex and other physiological stress parameters in many preliminary studies, even in those *with* a non-intervention control (see for examples Rupp et al., 2017 and Benke et al., 2021).

## Conclusion

This work is to be understood as a basic study of the CIPOS intervention, which aims to increase the state of knowledge about the efficacy and mechanisms of action of this therapeutically widely used method. Contrasting CIPOS with an exposition-only condition in a non-clinical study sample, changes in emotional reactivity during the confrontation with personally distressing affective scripts were investigated. Besides subjective measures of emotional valence and arousal, objective physiological measures (startle reflex as valence measure, SCL as arousal measure) were also recorded.

Overall, the study demonstrates the efficacy of CIPOS in reducing the distress caused by stressful autobiographic memories at both subjective and objective levels, as intended by Jim Knipe with his approach more than a decade ago. Moreover, the considerate and resource-oriented experience of stress reduction can be understood as a mastery experience that encourages and empowers patients to engage in subsequent trauma confrontational treatment. The importance of safety perception in trauma healing may be the reason why CIPOS has the potential for becoming a long-lasting procedure in mental health by addressing and promoting these issues. Moreover, as safety is a kind of “trans-approach” construct, presented in both psychodynamic-oriented interventions (see for example all the derivations from attachment theory) and CBT-oriented ones, CIPOS could be integrated as a basic intervention that fits different superordinate therapy concepts.

Since this work is the first of its kind, further studies must be undertaken to replicate the witnessed effects in healthy individuals, as well as investigate effects in patients with PTSD.

## Data availability statement

The raw data supporting the conclusions of this article will be made available by the authors, without undue reservation.

## Ethics statement

The studies involving human participants were reviewed and approved by local ethic committee of the faculty of medicine Justus Liebig University. The patients/participants provided their written informed consent to participate in this study.

## Author contributions

MS and VP: conceptualization, methodology, original draft preparation, visualization, project administration, and obtained ethical approval. GS: software, data curation, and methodology. FZ: data acquisition. BH and OT: writing – review and editing. MS: supervision. All authors contributed to the article and approved the submitted version.

## Conflict of interest

The authors declare that the research was conducted in the absence of any commercial or financial relationships that could be construed as a potential conflict of interest.

## Publisher’s note

All claims expressed in this article are solely those of the authors and do not necessarily represent those of their affiliated organizations, or those of the publisher, the editors and the reviewers. Any product that may be evaluated in this article, or claim that may be made by its manufacturer, is not guaranteed or endorsed by the publisher.
